# Molecular insights into chronotype and time-of-day effects on decision-making

**DOI:** 10.1038/srep29392

**Published:** 2016-07-08

**Authors:** Krista K Ingram, Ahmet Ay, Soo Bin Kwon, Kerri Woods, Sue Escobar, Molly Gordon, Isaac H. Smith, Neil Bearden, Allan Filipowicz, Kriti Jain

**Affiliations:** 1 Department of Biology, Colgate University, Hamilton, NY, USA; 2 Department of Mathematics, Colgate University, Hamilton, NY, USA; 3 Johnson Graduate School of Management, Cornell University, Ithaca, NY, USA; 4 INSEAD Business School, 1 Ayer Rajah Avenue, Singapore 138676; 5 IE Business School, María de Molina, Madrid, Spain 11 28006.

## Abstract

Recent reports highlight that human decision-making is influenced by the time of day and whether one is a morning or evening person (i.e., chronotype). Here, we test whether these behavioral effects are associated with endogenous biological rhythms. We asked participants to complete two well-established decision-making tasks in the morning or evening: the matrix task (an ethical decision task) and the balloon analog risk task (BART; a risk-taking task), and we measured their chronotype in two ways. First, participants completed a self-report measure, the Horne-Östberg Morningness-Eveningness Questionnaire (MEQ). Second, we measured the expression of two circadian clock-regulated genes—*Per3* and *Nr1d2—*from peripheral clock cells in participants’ hair follicle samples. Using a cosinor model, we estimated the phase of the peripheral clock and assigned RNA chronotypes to participants with advanced (larks) or delayed (owls) phases. The behavioral data were analyzed independently for self-reported (MEQ) and RNA-based chronotypes. We find that significant chronotype and/or time-of-day effects between larks and owls in decision-making tasks occur only in RNA-based chronotypes. Our results provide evidence that time-of-day effects on decision-making can be explained by phase differences in oscillating clock genes and suggest that variation in the molecular clockwork may influence inter-individual differences in decision-making behavior.

Recent studies have highlighted time-of-day effects on human behavior and cognition, including ethical decision making^1^. ‘Morning-morality’ and similar time-of-day effects can be explained by the depletion of self-regulatory resources throughout the day^1^,^2^,^3^. However, it has been shown that these effects can be moderated by an individual’s chronotype—a preference for activity early in the day or late in the evening—based on self-reported morningness-eveningness questionnaires (MEQs): eg. morning-type individuals (‘larks’) have been shown to cheat more in the evening, and evening-type individuals (‘owls’) have been shown to cheat more in the morning^4^. The underlying assumptions in the interpretation of these data are that the internal rhythms of larks and owls differ and behavioral changes occur when an individual is making decisions during particular physiological states (peaks or troughs in arousal) within the context of their personal daily rhythm.

Sleep-wake cycles and daily rhythms in physiological arousal are regulated by two mechanisms: circadian drive and homeostatic drive^5^,^6^. Under circadian drive, states of arousal fluctuate according to a daily rhythm that is dictated by an internal molecular pacemaker^7^,^8^. Variation in the timing of the daily endogenous rhythms between individuals results in diverse chronotypes and is predicted to lead to distinct behavioral responses to stimuli depending on the time of day^9^. Interactions between chronotype and time of day, so-called synchrony effects^9^, occur when the timing of optimal behavioral responses parallels the phase of circadian arousal across individuals—e.g., morning-type individuals perform better at cognitive tasks in the morning and vice versa for evening-type individuals. In contrast, under homeostatic drive, sleep propensity increases and physiological arousal decreases with diminishing energy stores throughout the day^7^. In lay terms, under homeostatic drive, energy levels decrease as a day progresses, whereas under circadian drive, energy levels peak at different times of the day depending on a person’s chronotype. Daily patterns of arousal (and downstream effects on performance of cognitive, emotional and attentional tasks) therefore likely depend on the interplay between these two forces.

There is a substantial body of literature exploring the association of chronotype, or diurnal preference, with human behavior and performance, with strong effects seen particularly in situations involving complex cognitive processing and executive function^9^,^10^,^11^. These studies report significant time of day effects on behavior due to multiple factors: age-related developmental changes from adolescence to adulthood^12^,^13^,^14^, significant behavioral differences between extreme chronotypes (larks and owls)^15^,^16^,^17^,^18^, and the difficulties people have in adapting their behavior to shifts in circadian timing^19^,^20^,^21^,^22^. The most widely used method for measuring chronotype in these studies is the Morningness-Eveningness Questionnaire (MEQ), designed by Horne and Östberg^23^. The MEQ and its variations measure self-reported preferences for sleep and lifestyle patterns, including timing of peak activity and alertness during the day, and timing of the sleep-wake cycle. Self-reported chronotypes are associated with physiological factors, most notably body temperature and melatonin production^24^,^25^,^26^,^27^,^28^,^29^,^30^, and studies have linked chronotypes to circadian-related gene expression in both *in vitro*^31^,^32^,^33^ and *in vivo* studies^24^,^34^,^35^,^36^,^37^. However, non-physiological factors may also influence the effect of chronotype on human behavior, including personality traits and emotional states^38^,^39^,^40^. Here, we explore the associations between natural variation in the timing of the molecular clock, self-reported chronotype, and individual performance on decision-making at different times of day.

Until recently, it has been difficult to study the biological basis of chronotype effects on behavior. The internal physiological rhythms that dictate an individual’s chronotype or diurnal preference are controlled primarily by the central circadian pacemaker that is located in the suprachiasmatic nuclei (SCN) region of the hypothalamus^41^. Oscillations of core clock genes, operating in negative feedback cycles, provide the daily rhythm to the central clock and regulate downstream physiological and metabolic processes via direct signaling from the SCN and via the signaling of peripheral clocks^42^. The central clock can be entrained by external cues (i.e light/dark cycles)^43^ and acts to synchronize the numerous peripheral clocks that reside in many bodily tissues—including vital organs, skin and hair follicles—to changes in the environment. Peripheral clocks themselves can also be regulated by exogeneous environmental or physiological cues^44^, so molecular oscillations in peripheral clocks act as a proxy, but not a direct measure, of the central oscillator. In order to understand how differences in the molecular clockwork between individuals are associated with chronotype, recent studies have successfully utilized peripheral clock tissues to measure oscillations in circadian gene expression *in vivo* and under real-life conditions^24^,^37^,^45^. Contrary to the rapid progress we have made in understanding circadian clock function on a molecular level, we know relatively little about how differences in the timing or phase of oscillations in the molecular clockwork affect human behavior and decision-making processes.

In this study, we test whether the interaction between chronotype and time-of-day affects decision-making in a broad sense and whether there is a biological basis to this behavioral interaction. Undergraduate students were asked to complete a self-report MEQ survey and to participate in two computer-based decision-making tasks. One task replicates, in part, the previous morning morality studies using a matrix-solving paradigm^46^, and the second task, the Balloon Analog Risk Task (BART)^47^, represents a well-documented measure of risky decision-making. There was no significant correlation between performance on the BART and matrix tasks (r^2^ = 0.07, F = 0.022, p = 0.883) and thus the results represent two independent tests of chronotype by time-of-day interactions on decision-making. From the MEQ survey, we determined the chronotype of participants; individuals that scored high on the survey (score > 59) were designated morning-types or MEQ-larks, and individuals that scored low on the survey (score < 41) were designated evening-types or MEQ-owls^23^.

To assess biological phase differences in circadian rhythms of participants, we measured oscillations in the expression of circadian clock genes in hair follicle cells^48^,^49^. Hair follicle peripheral clocks provide a non-invasive, real-time, *in-vivo* method of monitoring the circadian rhythms of participants^37^,^45^. Previous studies have described a method for estimating the phase of a cosinor curve in circadian gene oscillation using three time-points^37^,^45^. We use simulated data to validate this method and to determine the optimal three time-point sampling scheme. We measure the expression levels of two clock-related genes (*Per3*, *Nr1d2*) at three time points separated by 8 hours for participants with extreme lark or owl MEQ chronotypes and calculate phase differences in circadian clock gene oscillations for these individuals relative to a control group of participants of intermediate MEQ chronotypes. From this subset of the data, we designate RNA larks (individuals with advanced phases relative to intermediate chronotypes) and RNA owls (individuals with delayed phases relative to intermediate chronotypes). We then compare the behavioral results of the cheating and risky decision-making tasks from MEQ-based chronotypes to behavioral results from RNA-based chronotypes. We test the novel hypothesis that the behavior of self-reported, MEQ chronotypes parallels the behavior of RNA-based chronotypes defined by phase differences in circadian gene oscillations. Drawing from previous research, we expect to find synchrony effects due to circadian drive and significant effects of chronotype and time of day in both ethical and risky decision-making tasks. We also predict that chronotype and time of day effects will be more pronounced in larks as homeostatic drive will produce additive effects in lark behavior and opposing effects in owl behavior.

## Results

### Ethical Decision-Making

**Figure 1:**
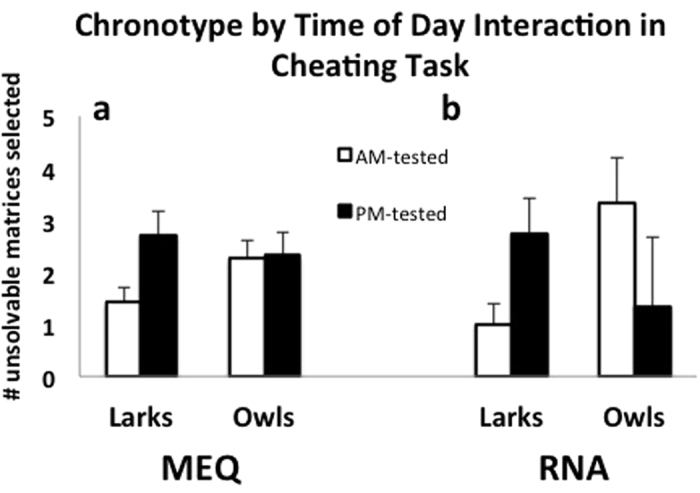
Chronotype by time of day interaction from matrix task results (±SE) for MEQ-based (**a**) and RNA-based chronotypes (**b**). Y-axis represents the number of matrices incorrectly reported with solutions. There were no significant main effects of chronotype or time of day or interactions for MEQ-chronotypes. For RNA-based chronotypes, there are no significant main effects for chronotype or time of day, but there is a significant interaction effect (F(1,20) = 5.24, p = 0.04, η^2^ = 0.21; n = 16 larks, 8 owls).

For the cheating measure, there are no significant main effects for self-reported MEQ larks and owl chronotypes (F(1,68) = 0.12, p = 0.73, η^2^ < 0.01), time of day the task is performed (F(1,68) = 2.46, p = 0.12, η^2^ = 0.03), or the interaction between MEQ chronotype and time of day (F(1,68) = 0.89, p = 0.35; η^2^ = 0.01; n = 30 larks and 42 owls; Fig. 1a, Table 1). We roughly replicated the results of Kouchaki & Smith^1^ with individuals tested in afternoon cheating more than those tested in morning (PM participants: *M* = 2.52, SE = 0.09, n = 45; AM participants: *M* = 1.84, SE = 0.07, n = 27; F(1,70) = 3.24, p = 0.08, η^2^ = 0.04). When we include gender as a covariate in the MEQ model, there were no significant main effects of gender on cheating (F(1,66) = 1.98, p = 0.17, η^2^ = 0.03), and the interactions of gender with MEQ-chronotype and time of day were not significant.

When chronotype is predicted from circadian clock phase using clock gene mRNA levels, a significant interaction effect is observed and RNA larks (individuals with advanced phases) and RNA owls (individuals with delayed phases) behave differently at different times of the day (Fig. 1b, Table 1). For RNA-based chronotypes, there are no significant main effects for chronotype (F(1,20) = 0.31, p = 0.58, η^2^ = 0.02) or time of day (F(1,20) = 0.02, p = 0.88, η^2^ < 0.01), but there is a significant interaction effect (F(1,20) = 5.24, p = 0.04, η^2^ = 0.21; n = 16 larks, 8 owls). RNA larks cheat three times more in the evening than in the morning (F(1,14) = 5.95, p = 0.03, η^2^ = 0.30), and the opposite trend is seen in RNA-owls, who tend to cheat more in the morning (F(1,6) = 3.06, p = 0.14, η^2^ = 0.34). With gender included as a covariate in the RNA model, there were no significant main effects of gender on cheating (F(1,18) = 2.92, p = 0.11, η^2^ = 0.14) and the interactions of gender with RNA chronotype and time of day were not significant.

### Risky Decision-Making

**Figure 2:**
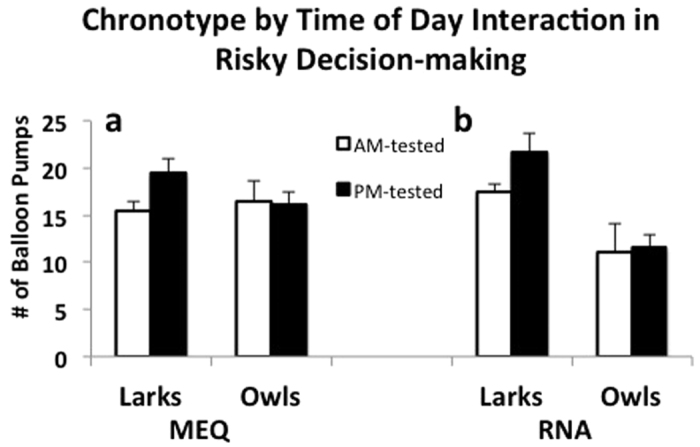
Chronotype by time of day results (±SE) from Balloon Task for MEQ-based (**a**) and RNA-based chronotypes (**b**). Y-axis represents the average number of balloon pumps per trial (n = 15 trials). There were no significant main effects of chronotype or time of day or interactions for MEQ-chronotypes. For RNA-based chronotypes, we find a main effect on chronotypes (F(1,20) = 18.36, p = 0.001, η^2^ = 0.48; n(larks) = 16, n(owls) = 8); RNA larks take significantly more risks than owls as a group and these larks take significantly more risks when tested later in the day (F(1,14) = 7.40, p = 0.02, η^2^ = 0.34).

For the risk-taking measure, there are no significant main effects for MEQ chronotype (F(1,58) = 0.49, p = 0.49, η^2^ = 0.01), time of day the task is performed (F(1,58) = 1.22, p = 0.27, η^2^ = 0.02), or interaction between MEQ chronotype and time of day (F(1,58) = 1.60, p = 0.21, η^2^ = 0.03; Fig. 2a, Table 2). When we include gender as a covariate in the MEQ model, there were no significant main effects of gender on risk-taking (F(1,56) = 0.57, p = 0.45, η^2^ = 0.01 and the interactions of gender with MEQ-chronotype and time of day were not significant.

For RNA-based chronotypes in risky-decision tasks (Fig. 2b, Table 2), we find a main effect on chronotype; RNA larks take significantly more risks than owls with an average of 1.7 times more pumps per round of the balloon task (F(1,20) = 18.36, p = 0.001, η^2^ = 0.48; n(larks) = 16, n(owls) = 8) but there are no significant effects of time of day (F(1,20) = 1.55, p = 0.24, η^2^ = 0.07) or interaction effects of chronotype by time of day (F(1,20) = 0.98, p = 0.34, η^2^ = 0.05). RNA larks take 1.9 times more balloon pumps on average than RNA owls when tested in the evening (F(1,14) = 7.40, p = 0.02, η^2^ = 0.34). RNA owls do not show significant time-of-day effects on risky decision-making (F(1,6) = 0.69, p = 0.83, η^2^ = 0.10). With gender included as a covariate in the model, there were no significant main effects of gender on risk-taking (F(1,18) = 0.12, p = 0.74, η^2^ = 0.01) and the interactions of gender with RNA chronotype and time of day were not significant.

### Sleep Quality

To test for effects of sleep quality, behavioral task data were compared between participants with good quality sleep (lower quartile of PROMIS scores) and poor quality sleep (upper quartile of PROMIS scores). There was no significant main effect of sleep quality or time of day on behavior for either decision-making task (matrix task: F(1,68) = 0.01, p = 0.94, η^2^ = 0.00; balloon task: F(1,58) = 0.59, p = 0.45, η^2^ = 0.01) and no significant interaction effects for either task.

## Discussion

Our results demonstrate that chronotype and time-of-day effects on decision-making are more pronounced in RNA-based chronotypes relative to MEQ-based chronotypes. The fact that individuals with measurable differences in the phase of oscillating clock genes show more pronounced behavioral effects suggests that differences in the timing of circadian drive may influence individual decision-making. Using two independent tests of decision-making, we find significant behavioral differences with chronotype and time-of-day in RNA-based chronotypes in both measures, but the associations between these factors and behavior differ across the two cognitive tasks.

In the matrix task, the interaction between chronotype and time-of-day significantly influenced behavior, suggesting a synchrony effect in ethical decision-making. The morning morality effect^1^, the tendency for people to act more ethically in the morning than in the evening, is attributed to homeostatic processes that regulate energy expenditure throughout the day; i.e. people are worn out at the end of the day^50^,^51^ and lack the energy to self-regulate. Our data roughly replicate the findings of the Kouchaki & Smith^1^ study that individuals cheat more often on the matrix task in the evening. The importance of chronotype was proposed in Gunia and colleagues^4^; they concluded that chronotype morality effect was a better predictor of ethical behavior than time of day alone. Our data supports the chronotype morality effect with a significant chronotype by time of day interaction for RNA-based chronotypes but not self-reported chronotypes. The large effect size of the interaction in RNA-based chronotypes further suggests that this effect is driven by phase differences in the endogenous oscillation of clock genes.

Less empirical evidence is available in the literature on chronotype effects in risky decision-making, although the expectation of tasks involving such effortful executive function mirrors that of ethical decision-making, with a synchrony effect of better performance at time of peak circadian arousal^11^,^14^. In our study of risky decision-making, we find a counterintuitive main effect that larks, as a group, take more risks than owls. In previous studies, morning types have been found to exhibit less propensity to take risks^52^,^53^. These studies used the DOSPERT^54^, a well-established self-report measure of intentions to take risks (i.e. “please indicate the likelihood that you would engage in the described activity or behavior”). But intentions to take risks are not the same as actual risk-taking behavior^47^. When Kilgore^18^ assessed intentions to take risks (using the DOSPERT) and actual risky behavior (using the BART) for morning and evening types, he found that evening types had a higher propensity to take financial risks, but there was no effect for actual risk-taking behavior on the BART task. We also measured risk-taking behavior using the BART and found that morning types took more risks than evening types and take increasing risks later in the day. The strong effect sizes of these results suggest that homeostatic drive may have a strong influence on risk measures in this study, causing larks to be more risk-prone than expected (in the evening).

Our findings lend further support to the additive energetic costs of homeostatic and circadian drives in larks; with depleted energy resources and low circadian drive in the evening, the ability of larks to self-regulate is diminished and they are more likely to make mistakes and take risks^50^,^55^,^56^. Alternatively, other physiological factors correlated with extreme circadian phenotypes may decrease risk-taking in owls relative to larks. Eveningness is associated with higher incidences of depression and anxiety^57^. Anxiety, the stable predisposition to perceive undetermined threats in the environment, is associated with lower risk-taking^58^,^59^. Under certain conditions, evening types might exhibit less risk seeking behavior, mediated by their higher levels of anxiety. The relationships between intended risks, actual risk-taking and chronotypes require further study.

One limitation in this study is that we did not have an independent measure of peak circadian arousal to directly compare circadian gene oscillations, arousal and behavior. In addition, we used only the Horne-Östberg MEQ to measure chronotype (in order to make direct comparisons to previous studies on chronotype and decision-making). A more recent method for measuring chronotype, the Munich Chronotype Questionnaire (MCTQ)^60^,^61^, also uses a self-report questionnaire on sleep and lifestyle patterns but includes a measure of the midsleep phase or mid-point of an individual’s sleep cycle and a measure of sleep debt. This allows for a measure of both the sleep-wake cycle and the phase angle of entrainment. We did include a measure of sleep quality (the PROMIS survey^62^) to control for the effect of poor sleep quality or sleep deprivation on the behavioral tasks as these tasks can be strongly influenced by sleep deprivation^18^. Sleep quality had no effect on behavior in this study.

In recent studies, chronotypes also appear to be correlated with the circadian period length *in-vitro*^31^,^32^,^63^,^64^ (but see^33^); morning-type individuals had generally shorter period lengths than evening-type individuals. However, the *in-vitro* period measured from disassociated peripheral tissues (e.g., in cultured skin cells) is not necessarily correlated with the *in-vivo* period of individuals, particularly those of extreme phenotypes^33^,^64^. Although the intrinsic circadian period is a critical factor in the pathophysiology of circadian-related sleep disorders, we were not able to measure this parameter in our study due to the mathematical limitations of estimating circadian phase from three data points. Further studies will be required to address whether an individual’s chronotype and decision-making behavior are associated with the *in-vitro* circadian period measured from peripheral hair follicles.

In this study, RNA-larks had phase advances (>1.5 hrs) and RNA-owls had phase delays (>1.5 hrs) relative to intermediate training subjects. This three-hour phase difference between morning-types and evening-types approximates the circadian phase differences documented in other chronotype studies comparing physiological rhythms (patterns of temperature oscillations or melatonin production)^24^,^27^,^28^,^29^,^36^,^65^ or molecular rhythms (circadian gene expression from peripheral blood cells, hair follicles, or fibroblast cultures of skin biopsies)^31^,^37^,^45^. Thus, three-hour phase differences in the molecular clockwork appear to be sufficient to influence cognitive and self-regulatory processing and to affect behavioral decision-making. These findings have significant implications for basic research on shift work and jet lag (i.e. optimizing schedules for important decision-making to minimize the effects of endogenous clock function), as well as everyday circumstances in which there might be a time lag between lifestyle and human performance or optimal decision-making.

## Methods

### MEQ Chronotype Analysis

Students from the undergraduate population of Colgate University (n = 139) participated in the study. All subjects completed the automated computer survey and genetic sampling (41 males, 98 females, age range 18–23). We recruited approximately half of the participants from an introductory biology course and other students responded to an open request for study participation; all students received payment and/or course credit for participation. The demography of the Colgate University undergraduate population is: 69% Caucasian, 10% Latino, 9% Asian, 5% African American, 56% female, and mixed socio-economic status. Our participants were sampled across this ethnic and socio-economic diversity. Our sampling was not discipline specific. An automated survey, including the Horne-Östberg Morningness-Eveningness Questionnaire (MEQ), was administered to each participant in the morning (AM) between 7:30–9 AM or in the evening (PM) between 4:30–6 or 8–10 PM. Because there was no difference between the afternoon and evening data, we collapsed these two groups into one evening group. The MEQ consists of 19 questions that assess diurnal preference (i.e. timing of daytime activities, sleeping habits, hours of peak performance, times of maximum alertness, etc.). Individuals with high scores represent moderate (>59) or extreme (>70) morningness chronotypes while individuals with low scores represent moderate (<41) or extreme (<30) eveningness chronotypes. The correlation between MEQ scores and sleep-wake cycles has been validated in numerous previous studies^66^,^67^. 71 of these individuals scored as moderate or extreme chronotypes; 29 were identified as ‘morning’ chronotypes and 42 as ‘evening’ chronotypes. 68 individuals had intermediate scores and were designated as ‘neither’. All methods were developed in agreement with the Declaration of Helsinki; procedures and consent forms were approved by the Institutional Review Board at Colgate University (#FR-F13–07). All methods were carried out in accordance with the approved guidelines. Informed consent was obtained from all individuals before samples were taken.

### Decision-making Task Analysis

#### Risk-taking task

We measured actual risk-taking behavior using a computer based behavioral risk measure, the BART (Balloon Analog Risk Task) to assess risky behaviors. In each round, participants earn money each time they pump up a balloon, but lose all the money from the round if the balloon bursts before they take their payout. We picked one person at random from the study population, and paid them their winnings on the BART. This is a robust measure of risk-taking behavior, as risk is assessed with 15 trials.

#### Ethical Decision-making

Participants also completed a computerized matrix task (similar to^1^) in which they earned 25¢ for each correctly solved matrix. Participants were presented with 20 matrices containing 12 three-digit numbers (e.g. 3.69). Participants were asked to indicate only whether they found a matching pair (i.e., two numbers that would add up to 10) in each matrix within 15 seconds (they are not required to identify the two numbers. Twelve of the matrices were solvable (i.e., contained two numbers that summed to 10), and the remaining 8 were unsolvable (i.e., did not contain two numbers that summed to 10). Thus, this task allowed us to gauge whether someone cheated; i.e. found matrices that summed to 10 when they didn’t exist.

### RNA Analysis

We collected 15–20 hair follicles from 38 participants at three time points spaced 8 hours apart (8:00, 16:00, 24:00). Samples were stored in RNAlater^®^ at −80 °C prior to analysis. RNA was purified from hair follicles by using an RNeasy Micro purification kit according to the protocol provided by Qiagen. The purified RNA was converted to cDNA via rt-PCR (TaqMan Gold rt-PCR, ABI). Expression of clock genes was measured via quantitative PCR on an ABI 7900HT instrument (Applied Biosystems). qPCR analyses were performed in triplicate for two principal clock genes (*Per3*, *Nr1d2*) and the control gene, *18S*. The quantification of relative mRNA levels and standard errors was calculated using the standard curve method (ABI User Bulletin #2).

### Validation of Model for Phase Prediction of Circadian Oscillation

A common method for estimating the phase of circadian oscillations utilizes a cosinor curve analysis^68^. Akashi and colleagues^45^ modified this method for analysis of circadian gene expression data from three time-points. We used simulated data to validate this method with one or two genes and to determine the optimal three time-point sampling scheme.

#### Validation of three-point prediction

Ten training subjects with two gene expression data sets (*Per3, Nr1d2*) were generated using the equations below^45^.


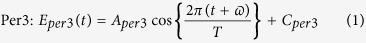



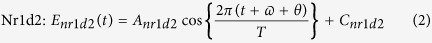


All parameters were randomly generated around the biologically realistic values used in previous studies^45^ with a standard deviation of 1. Amplitude (*A*) of each training subject was set ≈ 2, offset (*C*) ≈ 2, and period length (*T*) ≈ 24. Phase (*ω*) for *Per3* was set ≈ 0, and phase for *Nr1d2* was 2.2, based on the average phase difference between these two genes reported in Akashi *et al*.^45^. In our study, each training subject is allowed to have different amplitudes and offsets for the two genes. Mean amplitude, phase, offset, and period of these training subjects were then used to define the ‘standard curve.’

In addition to the ten training subjects, two gene expression data sets from ten test subjects (representing extreme owl chronotypes) were generated with the same parameter variation but with their phase for *Per3* set ≈ 3 and their phase for *Nr1d2* set ≈ 5.2 and with 5% biological noise (following Gaussian distribution) added to their expression data. For each test subject’s gene expression data, we extract the expression level at three different time-points. All possible combinations of three time-points over the span of 36 hours (1.5 cycles), which gave us 7770 combinations of three time-points ranging from [0 1 2] to [34 35 36], are considered in this study. For example, three data points at [8 12 16] represent the gene expression data of *Per3* and *Nr1d2* at 8am, 12pm, and 4pm from the test subject.

The standard curve created based on the average of our training subjects were fitted to the three data points of the test subjects using the parameter estimation method SRES (Stochastic Ranking Evolutionary Strategy)^69^. Because we want to predict the phase of our test subjects, phase of the standard curve is left flexible and the rest of the parameters of the standard curve (amplitude, offset, and period) are fixed. In other words, the standard curve can move only horizontally to fit the test data. The algorithm assumes the phase of *Nr1d2* to be 2.2 h larger than that of *Per3*^45^ and predicts the phase of *Per3* only. This was repeated 10 times with different sets of training and test subjects. Our result validated that it is possible to use only three time-points to estimate phase of one’s circadian rhythm and that certain three time-points work better than others and can guarantee a fairly accurate prediction.

#### Optimization of Sampling Time Points

To represent the three sampling time points that provide the most accurate phase prediction, we created a three-dimensional heatmap of all the parameters that predicted the phase with an error <0.5 hr using only one gene (Suppl. Fig.1). We repeated our simulation using two genes and created an equivalent heatmap. (Suppl. Fig. 2). Between the two heatmaps, we found 114 overlapping combinations that consistently gave us a good prediction of phase (Suppl. Fig. 3). For this study, we chose one optimal time-point combination [8 16 24], which consistently gave us accurate predictions.

### Prediction of Circadian Phase and RNA Chronotypes Analysis

To distinguish RNA-larks, owls, and intermediate subjects based on circadian phase, we applied our two-gene method to gene expression data collected from study participants at an optimal time-point combination of 8, 16 and 24 hrs. Our training data was obtained from 7 intermediate subjects (individuals with intermediate MEQ scores) using 2 genes and 8 gene expression collection time points, allowing us to have a robust standard curve. Using at least 4 subjects, we generated 64 different standard curves by bootstrapping across training subjects. Our test data was obtained from 27 subjects with moderate or extreme MEQ scores using 2 genes and 3 data points. Using each standard curve, we estimated the phase for each test subject using our two-gene version of phase estimation and clustered them into three groups based on their average bootstrap phase value (average of phase estimates from each standard curve); RNA-larks (n = 16; *M*phase = 1.61, SE = 0.14) had phase advances (>1.5 hrs) and RNA-owls (n = 8; *M*phase = −2.11, SE = 0.31) had phase delays (>1.5 hrs) relative to intermediate training subjects. 71% of MEQ-larks and 89% of MEQ-owls also qualified as RNA-larks and owls respectively.

### PROMIS Sleep Quality Analysis

Sleep quality can also affect decision-making, so we also administered the PROMIS Sleep Disturbance Short Form survey to the participants^62^. This survey consists of 8 questions and is scored on a scale from 8 to 40 with lower scores representing good sleep quality and higher scores representing poor sleep quality. We re-analyzed the behavioral data using PROMIS scores (the higher and lower quartiles of sleep quality values recorded in this study) to test for an effect of sleep quality on behavior.

### Statistical Analysis

Differences in behavior on the matrix and BART task were tested using two-way ANOVAs with time of day and chronotype as factors and gender as a covariate. Separate analyses were performed for MEQ-chronotypes, RNA-based chronotypes, and PROMIS-types using SPSS.

## Additional Information

**How to cite this article**: Ingram, K. K. *et al*. Molecular insights into chronotype and time-of-day effects on decision-making. *Sci. Rep.* **6**, 29392; doi: 10.1038/srep29392 (2016).

## Supplementary Material

Supplementary Information

## Figures and Tables

**Table 1:** Cheating Measure in Ethical Decision-Making Task.

Group	Matrix Task			
	MEQ-based Chronotypes		RNA-based Chronotypes	
	*M*	SE	*M*	SE
*AM-tested*				
Lark	1.42	0.29	1.00	0.41
Owl	2.27	0.34	3.33	0.88
*PM-tested*				
Lark	2.71	0.46	2.75	0.67
Owl	2.32	0.46	1.33	1.33

*M* = average number of solutions found in matrices that did not have solutions per participant; SE = standard error.

**Table 2:** Risk Measure in BART Task.

Group	BART Task			
	MEQ-based Chronotypes		RNA-based Chronotypes	
	*M*	SE	*M*	SE
*AM-tested*				
Lark	15.52	0.92	17.38	0.91
Owl	16.44	2.13	11.11	2.02
*PM-tested*				
Lark	19.41	1.56	21.65	3.03
Owl	16.18	1.21	11.61	1.26

*M* = average number of balloon pumps per participant over 15 trials; SE = standard error.
