# Supplementary material for: Molecular insights into chronotype and time-of-day effects on decision-making
Source: Sci Rep. 2016 Jul 8;6:29392. doi: 10.1038/srep29392 (PMC4937423; doi:10.1038/srep29392)

**Supplementary Information for:**

**Molecular insights into chronotype and time-of-day effects on decision-making**

Krista K Ingram, Ahmet Ay, Soo Bin Kwon, Kerri Woods, Sue Escobar, Molly Gordon, Isaac H. Smith, Neil Bearden, Allan Filipowicz, Kriti Jain

**Suppl. Figure 1. Heat map of three time-point combinations optimized in one-gene model.** This heat map uses the data from our one-gene method (Per3). Each colored cube represents a combination of three time points. There were no duplicates and only those combinations with prediction error lower than or equal to 1 h were plotted. The darker colors indicate lower prediction error (and thus, better combinations). A total of 328 combinations are shown. The heat map showed two clusters of good combinations. It also provides evidence for the claim in Akashi et al, 2010 that the sampling time interval of 8h-8h gives the most accurate phase predictions.


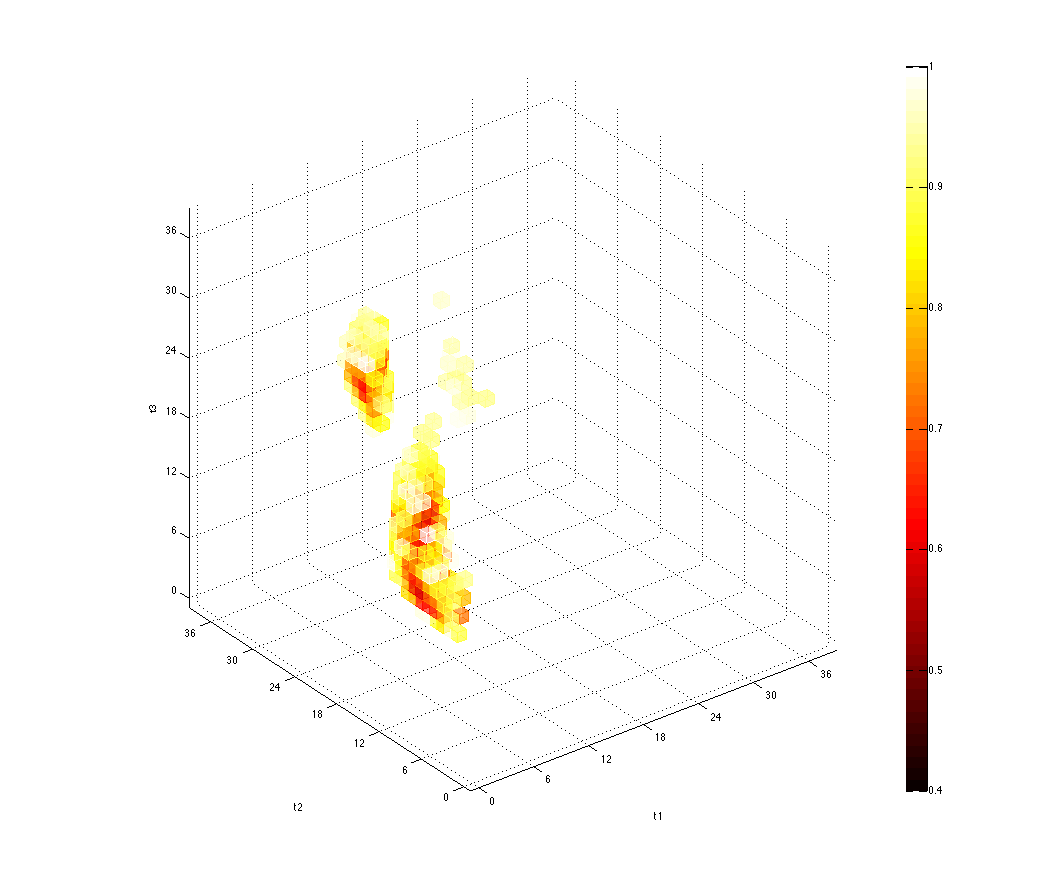


**Suppl. Figure 2. Heat map of three time-point combinations optimized in two-gene model.** This heat map uses the data from our two-gene method. This version fixes the phase of Nr1d2 to be 2.2 h larger than that of Per3. Only the phase of Per3 is predicted in this version. Only those that had prediction error for Per3 lower than or equal to 1 h were plotted. Each colored cube represents a combination of three time points. The darker colors indicate lower prediction error down to 0.4 h (and thus, better combinations). A total of 438 combinations are shown. The heat map showed two clusters of good combinations.


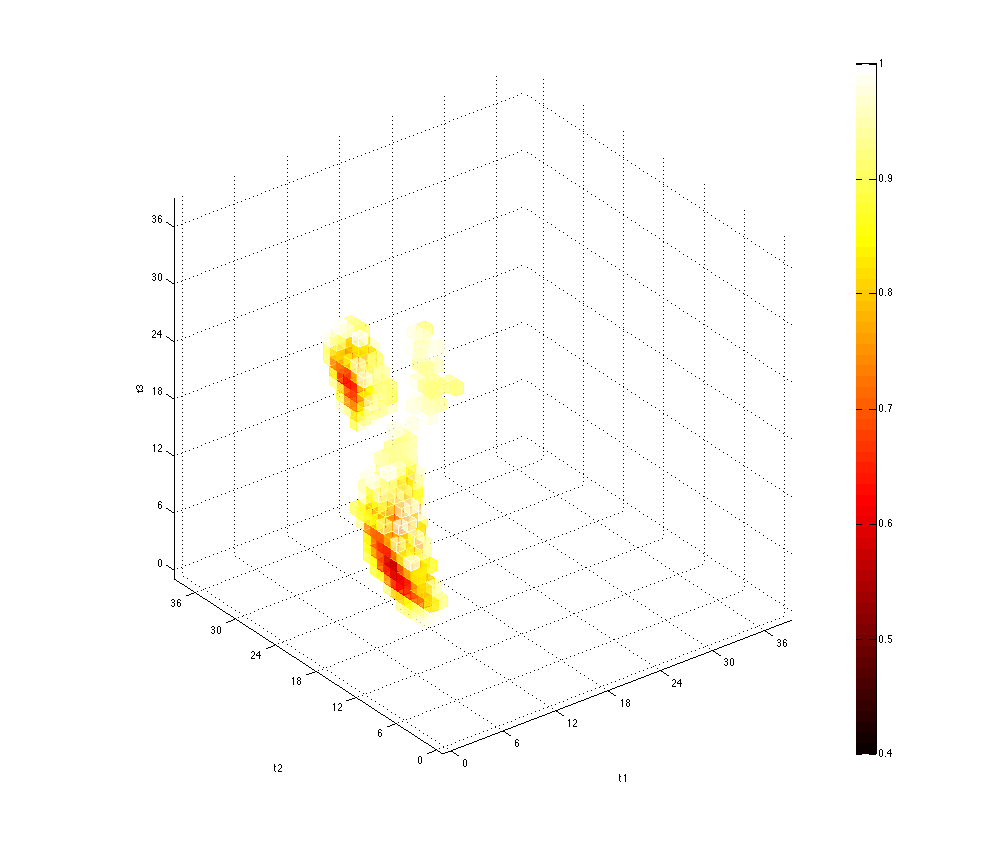


**Suppl. Figure 3. Heat map of overap in optimal three time-point combinations predicted from one- and two-gene models.** To compare our two-gene result with our one-gene result, we created a three-dimensional heatmap to determine overlap. The top 200 combinations from both versions were plotted based on how many times they passed our threshold. Green corresponds to the result with one gene, yellow corresponds to the result with two genes, and red corresponds to their overlap. The overlap between the one- and two-gene models provided 114 identical, optimal combinations. We chose one of these combinations, [8 16 24], for this study.


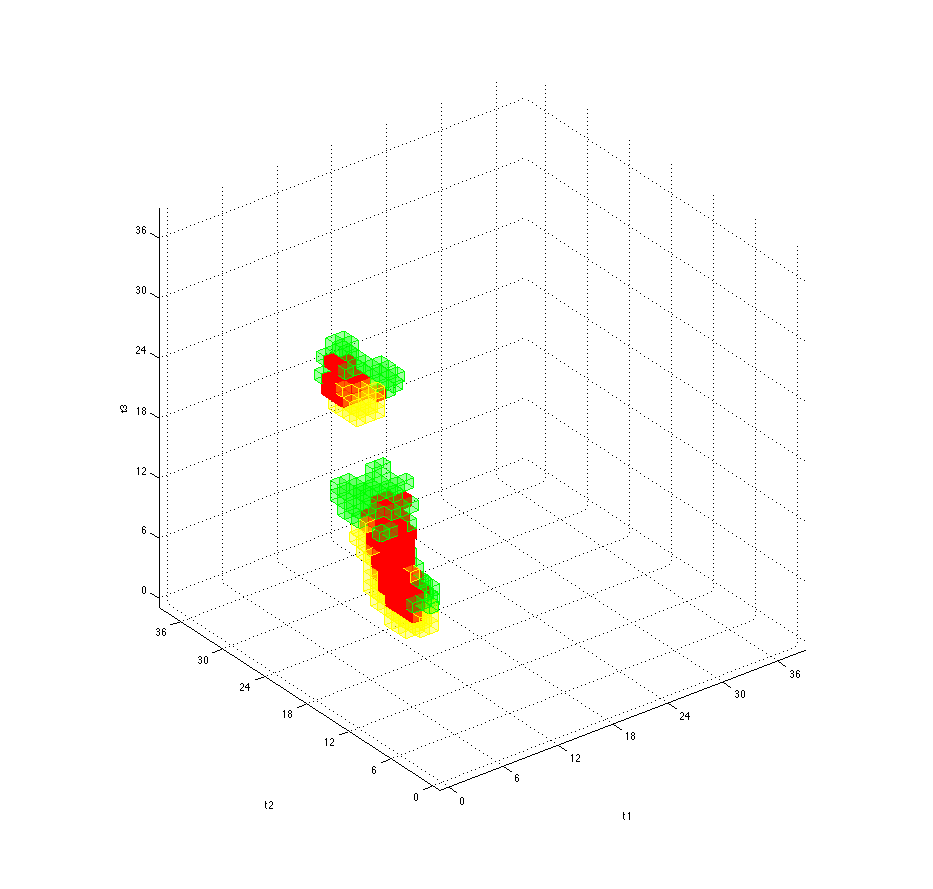

Supplement: Supplementary Information [file srep29392-s1.doc]
